# The economic burden of Diabetes Mellitus in Belgium from 2013 to 2017

**DOI:** 10.1093/eurpub/ckac131.146

**Published:** 2022-10-25

**Authors:** V Pinheiro, V Gorasso, B Devleesschauwer

**Affiliations:** 1 CINTESIS, Centre for Health Technology and Services Research, Porto, Portugal; 2 Public Health Unit, ACES Arco Ribeirinho, ARS LVT, Lisbon, Portugal; 3 Department of Epidemiology and Public Health, Sciensano, Brussels, Belgium; 4 Department of Public Health and Primary Care, Ghent University, Ghent, Belgium; 5 Faculty of Veterinary Medicine, Ghent University, Ghent, Belgium

## Abstract

**Background:**

Considering the growing prevalence of chronic disease and diabetes mellitus (DM) in Belgium, alongside population aging, insight into the economic burden of DM is essential for decision makers. To the best of our knowledge, there is no research on the subject in Belgium. Thus, our aim was to estimate the direct and indirect costs associated to DM in Belgium between 2013 and 2017.

**Methods:**

On a first phase, we performed a retrospective observational study, calculating the direct (i.e., ambulatory care, hospitalizations and medications) and indirect (work absenteeism, by multiplying mean daily wage and days absent from work) costs in the Belgian population with DM in 2013-2017. Data was retrieved from the Belgian Intermutualistic Agency (which manages compulsory health insurance) database and the Belgian Health Interview Survey database, namely DM prevalence, healthcare costs, days absent from work and sociodemographic and health factors. Subsequently, negative binomial regression models were used to assess the association of mean yearly costs to DM and adjustments for age, education level, physical activity, sugared drink consumption and body-mass index were included. Mean incremental costs were estimated through recycled predictions, considering the observed DM prevalence in Belgium in the study period and a counterfactual scenario with null prevalence.

**Results:**

We found a direct mean yearly incremental cost of €2 477 per DM patient, in Belgium, associated with age, low educational level and low physical activity. In the total Belgian population, the total yearly incremental healthcare cost of DM was €1.5 billion. Indirect yearly incremental cost of DM resulted to be not significantly different from the population without DM.

**Conclusions:**

DM has a major economic burden in Belgium, one that is expected to continue to rise in the future, alongside population aging. These results are essential for health planning and resource allocation.

**Key messages:**

• DM has a major economic burden in Belgium, especially when it comes to direct health expenditures with ambulatory care, hospitalizations and medications.

• Considering the growing prevalence of DM and population aging, these results are essential for health planning and resource allocation.

